# Prolonged Reactive Oxygen Species Production following Septic Insult

**DOI:** 10.4049/immunohorizons.2100027

**Published:** 2021-06-18

**Authors:** Isaac J. Jensen, Patrick W. McGonagill, Roger R. Berton, Brett A. Wagner, Elvia E. Silva, Garry R. Buettner, Thomas S. Griffith, Vladimir P. Badovinac

**Affiliations:** * Interdisciplinary Graduate Program in Immunology, University of Iowa, Iowa City, IA; † Department of Pathology, University of Iowa, Iowa City, IA; ‡ Department of Surgery, University of Iowa, Iowa City, IA; § Free Radical and Radiation Biology Program, Department of Radiation Oncology, University of Iowa, Iowa City, IA; ¶ Microbiology, Immunology, and Cancer Biology Ph.D. Program, University of Minnesota, Minneapolis, MN; ‖ Department of Urology, University of Minnesota, Minneapolis, MN; # Center for Immunology, University of Minnesota, Minneapolis, MN; ** Masonic Cancer Center, University of Minnesota, Minneapolis, MN; †† Minneapolis VA Health Care System, Minneapolis, MN; ‡‡ Department of Microbiology and Immunology, University of Iowa, Iowa City, IA

## Abstract

The dysregulated host response and organ damage following systemic infection that characterizes a septic event predisposes individuals to a chronic immunoparalysis state associated with severe transient lymphopenia and diminished lymphocyte function, thereby reducing long-term patient survival and quality of life. Recently, we observed lasting production of reactive oxygen species (ROS) in mice that survive sepsis. ROS production is a potent mechanism for targeting infection, but excessive ROS production can prove maladaptive by causing organ damage, impairing lymphocyte function, and promoting inflammaging, concepts paralleling sepsis-induced immunoparalysis. Notably, we observed an increased frequency of ROS-producing immature monocytes in septic hosts that was sustained for greater than 100 days postsurgery. Recent clinical trials have explored the use of vitamin C, a potent antioxidant, for treating septic patients. We observed that therapeutic vitamin C administration for sepsis limited ROS production by monocytes and reduced disease severity. Importantly, we also observed increased ROS production by immature monocytes in septic patients both at admission and ~28 days later, suggesting a durable and conserved feature that may influence the host immune response. Thus, lasting ROS production by immature monocytes is present in septic patients, and early intervention strategies to reduce it may improve host outcomes, potentially reducing sepsis-induced immunoparalysis.

## INTRODUCTION

There is a substantial health (affecting 1.7 million and killing 270,000) and economic (costing >20 billion dollars) burden imposed by sepsis on Americans annually (1). Over the past 30 years, the sepsis mortality rate has declined because of early intervention strategies aimed at reducing the severity of the septic cytokine storm (2, 3). This cytokine storm manifests through exaggerated production of cytokines, effector molecules, and reactive oxygen species (ROS) with the intent of rapidly controlling the systemic infection, but it also has the unintended consequence of organ damage (1, 4, 5). Yet, even as the cytokine storm resolves, patients continue to experience lasting (> five years) immunologic deficits, termed immunoparalysis, which reduce an individual’s capacity to control subsequent secondary infection (unrelated to the septic event) and cancer (2, 3, 6–9) or even undergo autoinflammatory activity (10). Consequently, the majority of sepsis-associated mortality is now dominated by late deaths from secondary infection and other morbid conditions due to immunologic impairment (11, 12).

Sepsis-induced immunoparalysis is characterized by severe transient lymphopenia (13–15), reduced capacity to produce effector molecules, reduced target recognition, and elevated inhibitory receptor expression by several immune cell populations (e.g., T cells, dendritic cells, and NK cells) (16–22). In addition to these intrinsic deficits of lymphocytes, extrinsic impairment is also present (e.g., capacity of endothelia to promote chemotaxis and suppression by monocyte-derived cells) (23–25). Notably, these impairments are also observed during “inflammaging,” wherein chronic, sterile, low-grade inflammation predisposes the host to age-related disease pathogenesis (26–28). The parallels between sepsis-induced and age-associated immunologic impairments suggest sepsis accelerates immunologic aging. Thus, the defects observed during sepsis-induced immunoparalysis may more accurately reflect alterations in immune cell function, due in part to chronic inflammation. Indeed, there is an increase in inflammatory monocytes following sepsis, suggesting ongoing inflammatory responses due to persistent cellular stress (29–33).

Monocytes are of particular importance in the postseptic environment, as they rapidly fill much of the space created during the sepsis-induced lymphopenia phase leading to splenomegaly (29). Specifically, a robust increase in Ly-6C^+^ monocytes, which tend toward an inflammatory phenotype, is seen. Alternately, myeloid-derived suppressor cells also increase in the postseptic environment and produce ROS to promote the chronic immunoparalysis state (33). In either scenario, the functional consequences of myelopoiesis and the proposed associated ROS production present a particularly potent mechanism by which these cells may contribute to the chronic immunoparalysis state.

Recently, the therapeutic administration of high-dose vitamin C, a potent antioxidant, has demonstrated some promise in improving sepsis patient survival (34, 35). Vitamin C therapy for sepsis is suspected to limit cellular stress and extraneous free radical production (36, 37). It is also possible that vitamin C therapy would further benefit the septic host by alleviating any ROS-mediated immune suppression (38). However, recent clinical trials and meta-analysis of clinical trials have indicated that vitamin C does not significantly improve septic patient survival or time of hospital stay (34, 39), although it may lead to short-term early improvements in survival (40). Regardless of these and other potential mechanisms, the extent to which vitamin C intervention has any benefit on the chronic immunoparalysis state that develops during a septic event remains under-evaluated. Using both a cecal ligation and puncture (CLP) murine model of sepsis and patient samples we describe a long-lasting elevation in the basal production of ROS by monocytes in previously septic hosts. The increase in ROS is attributed to a dramatic increase in immature CD11b^lo^ monocytes among the ROS-producing cells. Intriguingly, we also observed that therapeutic administration of vitamin C substantially reduces sepsis severity and the frequency of ROS-producing monocytes after sepsis resolution.

## MATERIALS AND METHODS

### Mice and infections

Inbred C57BL/6 (B6; Thy1.2/1.2) were purchased from the National Cancer Institute (Frederick, MD) and maintained in the animal facilities at the University of Iowa at the appropriate biosafety level. P14 C57BL/6-Tg mice were bred and maintained at the University of Iowa. Lymphocytic choriomeningitis virus (LCMV)–immune specific pathogen–experienced (SP_Exp_) mice were generated as previously described (17, 23, 41–45). Briefly, LCMV-Armstrong (LCMV-Arm) infections were administered by i.p. injection with 2 × 10^5^ PFU.

### Institutional setting and institutional review board approval

Patients were recruited at the University of Iowa Hospitals and Clinics, an 811-bed academic tertiary care center. Blood sample acquisition, patient data collection, and analysis were approved by the University of Iowa Institutional Review Board (identification number 201804822). Informed consent was obtained from patients or their legally authorized representatives.

### Sepsis patient selection and data collection

Subjects 18 y of age or older meeting sepsis-3 criteria for sepsis or septic shock (46) secondary to intra-abdominal infection, soft tissue infection, bloodstream infection, or pneumonia were enrolled. Exclusion criteria were infection requiring antibiotics in the past month, hospitalization for infection in the past year, and chemotherapy or radiation within the past year. Demographics and baseline characteristics, including age, gender, race, acute physiology and chronic health evaluation II (APACHE II) score, sequential organ failure assessment (SOFA) score, and presence of septic shock were collected. EDTA-treated blood samples were collected within 24 h of presentation and at 30 plus or minus 5 d.

### Healthy control patient selection and data collection

Healthy volunteers 25 to 80 y of age were recruited from University of Iowa faculty, staff, and graduate/professional students. Exclusion criteria were signs or symptoms of active infections, infection requiring antibiotics within the past month, infection requiring hospitalization in the past year, and chemotherapy or radiation in the past year. Demographic data, including age, gender, and race were collected. EDTA-treated blood samples were collected at a visit to our research clinic.

### CLP model of sepsis induction

Sham and CLP surgeries were performed as previously described (47, 48). Briefly, mice were anesthetized with ketamine/xylazine (Office of Animal Resources, University of Iowa), the abdomen was shaved and disinfected with Betadine (povidone-iodine; Purdue Products), and a midline incision was made. The distal third of the cecum was ligated with PERMA-HAND Silk (Ethicon), punctured once using a 25-gauge needle, and a small amount of fecal matter was extruded. The cecum was returned to abdomen, the peritoneum was closed with 641G PERMA-HAND Silk (Ethicon), and the skin was sealed using surgical Vetbond (3M). Following surgery, 1 ml of PBS was administered s.c. to provide postsurgery fluid resuscitation. Lidocaine was administered at the incision site, and flunixin meglumine (Phoenix Scientific) was administered for postoperative analgesia. This procedure created a septic state characterized by loss of appetite and body weight, ruffled hair, shivering, diarrhea, and/or periorbital exudates with 0–10% mortality rate. Sham mice underwent identical surgery, excluding CLP.

### Evaluation of disease severity

Disease severity for specific pathogen–free (SP_Free_) mice was rated as follows: 1) healthy; 2) ruffled fur; 3) ruffled fur and reduced mobility; 4) ruffled fur, reduced mobility, and cold to the touch; and 5) moribund/dead. Clinical signs of disease for SP_Exp_ mice were rated as follows: for grooming, 0) normal, 1) fur that has lost shine/become matted (dusty), 2) fur becomes erect or bristling (ruffled); for mobility, 0) mobile without stimulation (normal), 1) mice are less responsive/mobile to stimuli (reduced), 3) mice are unresponsive to stimuli (immobile); for body position, 0) body is fully extended (normal), 1) back is arched/curved (hunched), 2) laying on side at rest (on side); and for food intake, 0) normal food intake has been adjusted, to allow for surgery-specific weight loss to be mitigated because of minimal weight loss that occurs in sham controls (0–9.9%), 1) moderate weight loss (10–14.9%), 2) severe weight loss (15–19.9%). After giving one score for each category, the sum of all categories indicates disease score. Dead mice are given the highest score (8) on the day of death and thereafter removed from scoring. Healthy scores range from 0 to 2; moderate disease scores range from 3 to 5; and severe disease scores range from 6 to 8.

### Murine cell isolation

Single-cell suspensions from spleens were generated after mashing tissue through 70-μm cell strainer without enzymatic digestion.

### Human cell isolation and cryopreservation

Human cell isolation was as adjusted from the previously described methodology (49). Briefly, whole blood was centrifuged, and serum was removed. RBC lysis buffer was then added to the cell pellet and rested for 5 min at room temperature. Cells were again centrifuged, and supernatant was removed. Process was repeated one to two additional times. Cells were washed with PBS three times before being counted and resuspended in cell freeze media (90%FCS, 10%DMSO) and then stored at −80°C. When ready for use, frozen cells were rapidly thawed and placed into warmed complete media. Cells were then washed three times with warmed media, and aggregates were filtered prior to use.

### Adoptive transfer of P14 cells

Adoptive transfer (AT) of TCR-transgenic CD8 T cells was performed as previously described (9). Briefly, a small amount of blood was collected from Thy1.1/Thy1.1 P14 mice. RBCs were lysed, and the frequency of naive CD8 T cells was determined by flow cytometric analysis of a portion of the samples. Remaining cells were then enumerated and then adjusted to transfer 5 × 10^3^ naive CD8 T cells per mouse. Cells were transferred via retro-orbital injection.

### Flow cytometry and intracellular protein detection

Flow cytometry data were acquired on a FACSCanto (BD Biosciences, San Diego, CA) and analyzed with FlowJo software (Tree Star, Ashland, OR). To determine expression of cell surface proteins, mAb were incubated at 4°C for 20–30 min, and cells were fixed using BD Cytofix/Cytoperm solution (BD Biosciences). The following anti-mouse mAb clones were used: CD3 (17A2; BioLegend), CD11a (M17/4; BioLegend), Ly-6C (AL-21; BD Biosciences), KLRG1 (2F1; BioLegend), CD127 (SB/199; eBioscience), CD8a (53-0.67; BioLegend), Thy1.1 (HIS51; eBioscience), CD11b (M1/70; eBioscience), F4/80 (BM8; eBioscience), Ly-6G/C (RB6-8C5; BioLegend). The following anti-human mAb clones were used: CD14 (61D3; Tonbo Biosciences) and CD11b (ICRF44; Tonbo Biosciences). ROS-producing cells were determined via a CellROX Deep Red Kit according to the manufacturer’s instructions (Thermo Fisher Scientific). Briefly, cells were labeled with CellROX Deep Red dye for 30 min at 37°C in complete media prior to Ab labeling.

### Vitamin C administration

Stock solutions of vitamin C (pH 7) were prepared in the Electron Spin Resonance Core Facility (University of Iowa) under argon and stored in screw-top sealed test tubes at 4°C. Ascorbate concentration was verified using ε_265_ = 14,500 M^−1^cm^−1^ (50). Vitamin C was administered (bolus, i.p.) at a dosage of 0.1 mg/1 g mouse every 12 h, beginning 6 h after surgery for the first 3 d and then every 24 h for the next 6 d. Vitamin C was diluted to a volume of 100 μL/mouse immediately prior to injection using dH_2_O. dH_2_O was used as control treatment.

### Statistical analysis

Unless stated otherwise, data were analyzed using Prism 8 software (GraphPad), using a two-tailed Student *t* test (for two individual groups; if unequal variance, a Mann–Whitney *U* test was used), one-way ANOVA with Bonferroni post hoc test (for >2 individual groups; if unequal variance, a Kruskal–Wallis with Dunn post hoc test was used), two-way ANOVA (for multiparametric analysis of two or more individual groups; pairing was used for samples that came from the same animal), and a Fisher exact test (for categorical data from two individual groups) with a confidence interval of >95% to determine significance (*p ≤ 0.05). Data are presented as SEM.

## RESULTS

### Sepsis leads to ROS-dependent tandem fluorophore degradation

**FIGURE 1.**
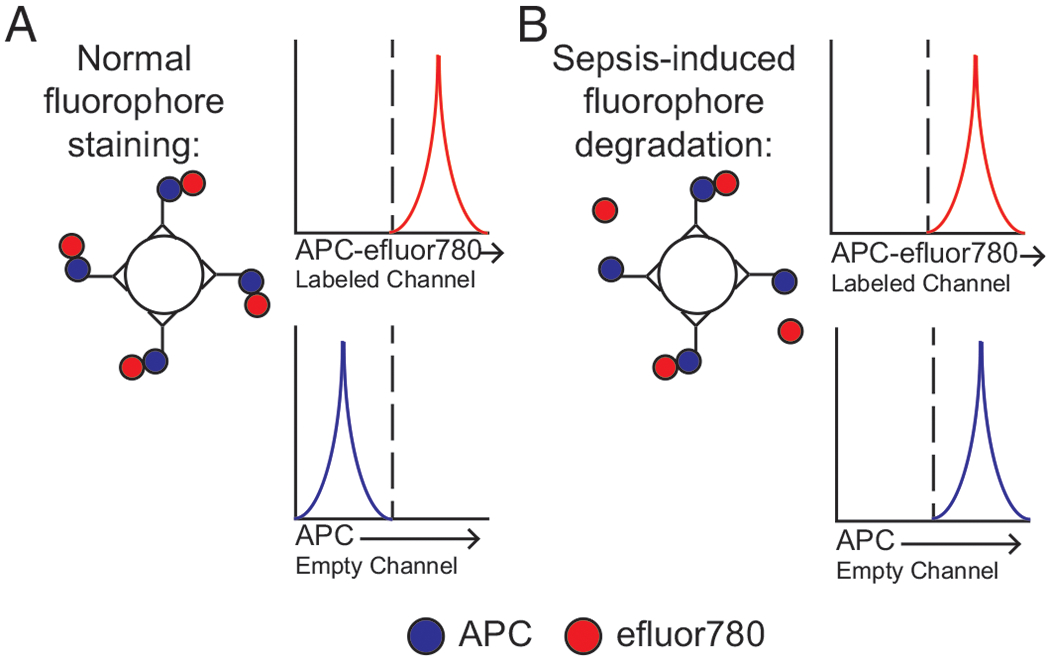
Model of sepsis-induced tandem fluorophore degradation. (**A)** Ideal model of nondegraded tandem fluorophore, wherein only signal for allophycocyanin (APC)–eFluor 780 but not allophycocyanin is detected. (**B**) Model of how sepsis-induced tandem fluorophore degradation leads to detection of both a signal for allophycocyanin–eFluor 780 and allophycocyanin. Figure is adapted from Ref. 51.

**FIGURE 2.**
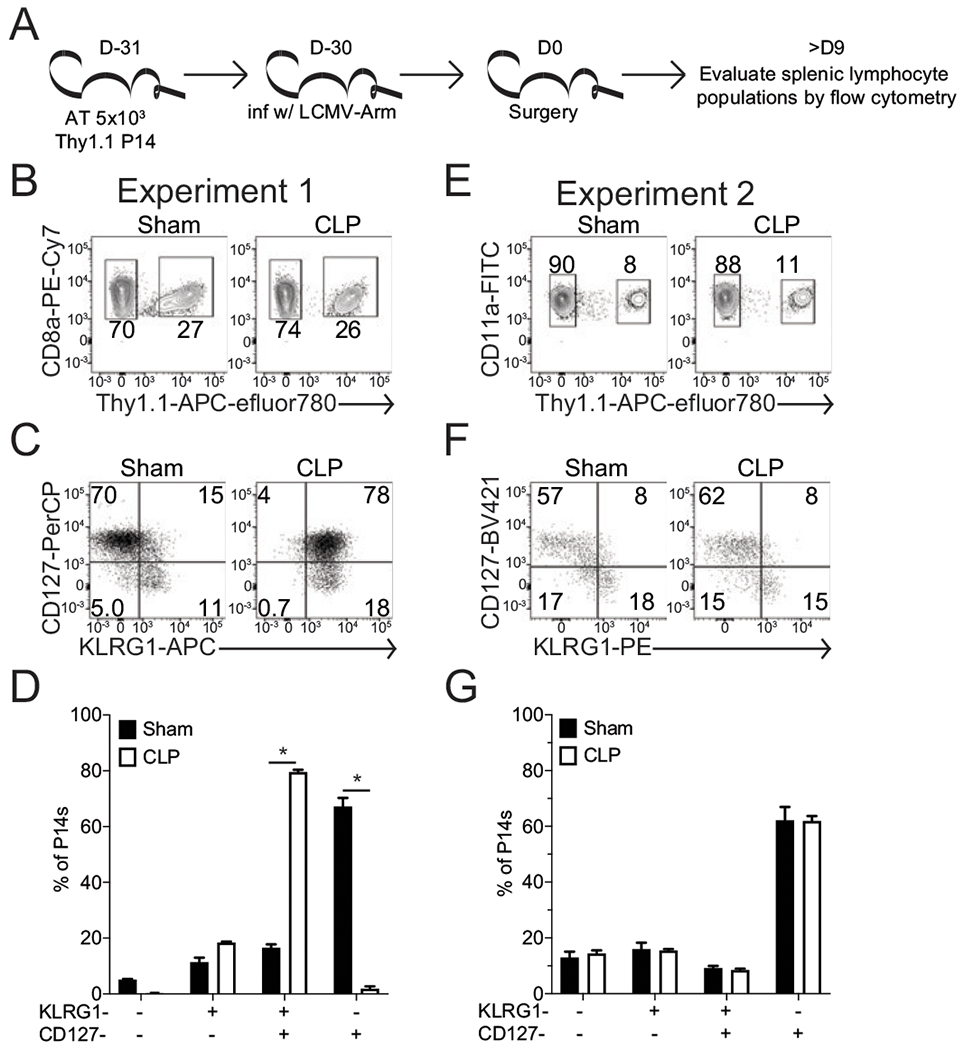
Sepsis leads to degradation of tandem fluorophores and impacts data interpretation. (**A**) Experimental design. C57BL/6 (Thy1.2) mice received an AT of 5 × 10^3^ naive TCR-transgenic P14 (Thy1.1) CD8 T cells by retro-orbital injection. The mice were infected 1 d later with LCMV-Arm (2 × 10^5^ PFU i.p.). Mice then underwent sham or CLP surgery 30 d postinfection. Memory P14 CD8 T cells were evaluated >10 d postsurgery (> 40 d postinfection). Experiment 1: (**B**) representative gating of P14 T cells identified by allophycocyanin (APC)–eFluor 780 (Thy1.1) expression in sham and CLP hosts. Representative gating (**C**) and cumulative data (**D**) of PerCP (CD127) and allophycocyanin (KLRG1) staining on P14 CD8 T cells in sham and CLP hosts. Experiment 2: (**E**) representative gating of P14 CD8 T cells identified by allophycocyanin–eFluor 780 (Thy1.1) expression in sham and CLP hosts. Representative gating (**F**) and cumulative data (**G**) of BV421 (CD127) and PE (KLRG1) staining on P14 cells in sham and CLP hosts. Data are representative of three repeated experiments with three to five replicates per group. Error bars indicate SEM. *p < 0.05.

Recently, we described the sepsis-induced degradation of tandem fluorophores mediated by ROS production [Fig. 1, adapted from Jensen et al. (51)]. A demonstration of how tandem fluorophore degradation can lead to the initial erroneous interpretation of flow cytometric data obtained from sham (control) and septic hosts is demonstrated in Fig. 2. Recently, Huggins et al. (52) demonstrated that immunologic experience influenced susceptibility to sepsis-induced mortality and the severity of the cytokine storm. Given that the majority of septic patients are elderly, with a high frequency of infection or vaccine-induced memory CD8 T cells, we had been seeking to better understand how sepsis may alter memory CD8 T cells number, function, and subset representation. Importantly, the number and type of memory CD8 T cells influence the capacity of memory CD8 T cells to provide protection during a pathogen rechallenge (41, 53), and sepsis leads to a dramatic numerical loss of memory CD8 T cells (9, 19, 54–56). Yet, how sepsis influences the loss and recovery of defined memory CD8 T cell subsets remains unknown (43, 44).

To investigate this important question, sham or CLP surgery was performed on mice bearing a defined population of memory CD8 T cells. To accomplish this, we used the well-established model of AT of naive TCR-transgenic CD8 T cells combined with experimental pathogen infection (9). Briefly, a small number of naive TCR-transgenic P14 CD8 T cells were transferred into congenically distinct naive C57BL/6 mice and then infected a day later with LCMV-Arm to elicit an Ag-specific response by the P14 CD8 T cells as well as endogenous GP_33_-specific CD8 T cells (41). At 30 d post-LCMV infection, an early memory timepoint, mice then underwent CLP surgery to induce acute peritonitis or sham control surgery (Fig. 2A). The extent to which sepsis influences the subset composition of virus-specific memory CD8 T cells was addressed by flow cytometry at >9 d after surgery (Fig. 2). Day >9 postsurgery was chosen, as it is a point at which numeric recovery of splenocytes is achieved following sepsis-induced lymphopenia. Importantly and relevant to this project, the error in interpretation of flow cytometric data occurred when the Thy1.1 P14 CD8 T cells were labeled by tandem dye (allophycocyanin–eFluor 780; Fig. 2B, 2E), and the degradation of the tandem fluorophore (i.e., allophycocyanin–eFluor 780) led to a “phantom” parent dye signal (in this case in the allophycocyanin channel). Because KLRG1 was labeled by allophycocyanin in the first set of experiments (Fig. 2C), the subsequent degradation of the Thy1.1-allophycocyanin-eFluor 780 caused an artificial increase in the percentage of KLRG1^+^ P14 CD8 T cells obtained from septic but not sham hosts (Fig. 2D). Because KLRG1 was labeled with a different fluorophore (PE) in the second set of experiments (Fig. 2F), the potential degradation of the allophycocyanin–eFluor 780 did not influence the perceived expression of KLRG1 (Fig. 2G). Thus, the KLRG1^+^CD127^+^ CD8 T cell population in the first experiment reflects the degradation of the tandem fluorophore (Fig. 2C, 2D). So, whereas sepsis did not alter the subset composition of memory CD8 T cell subsets early after sepsis induction, the fluorophore tandem degradation suggested increased ROS production >9 d after the septic insult (51). Based on these data, we refocused our attention to define the cellular source of this ROS production, its relevance to sepsis host recovery, and whether increased ROS production is a conserved feature with sepsis patients.

### Sepsis leads to a lasting increase in ROS-producing monocytes in mice

**FIGURE 3.**
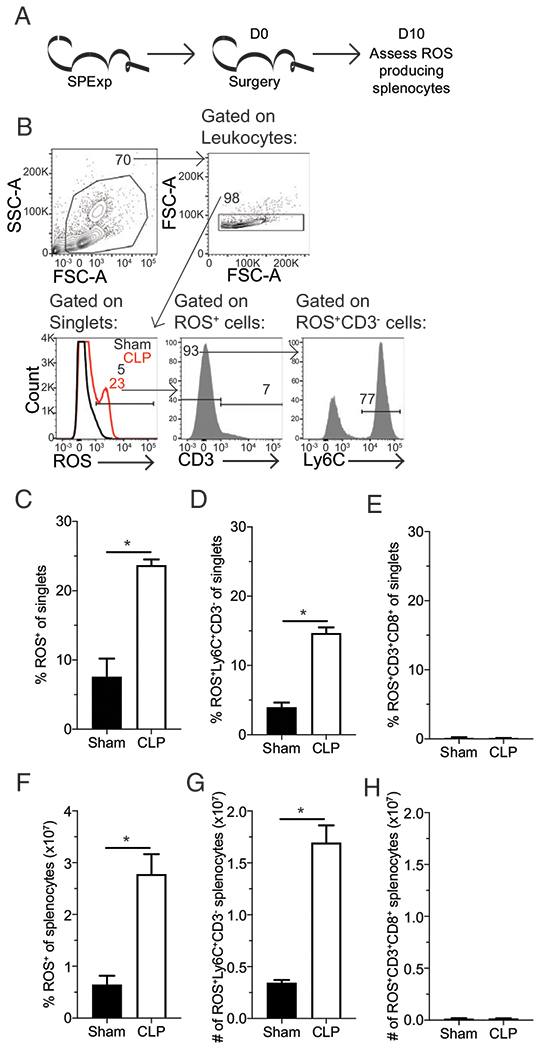
Sepsis leads to persistent ROS production by Ly-6C^+^ cells. (**A**) Experimental design. SP_Exp_ mice were generated by infection with LCMV-Arm (2 × 10^5^ PFU i.p.) and then underwent sham or CLP surgery 30 d postinfection. ROS-producing splenocytes were assessed 10 d later by flow cytometry. (**B**) Representative gating of ROS-producing cells. Frequency (**C**–**E**) and number (**F**–**H**) of (C and F) ROS-producing cells, (D and G) Ly-6C^+^CD3^−^ ROS-producing cells, and (E and H) CD3^+^ CD8α^+^ ROS-producing cells among splenocytes. Data are representative of three repeated experiments with two to three replicates per group. Error bars indicate SEM. *p < 0.05.

To maintain the relevance to immunologically experienced hosts, we continued to use mice that had undergone LCMV infection, in this study forward referred to as SP_Exp_ mice. To determine the cellular source of the ROS that is mediating the tandem fluorophore degradation seen in Fig. 2, SP_Exp_ mice underwent either sham or CLP surgery 30 d postinfection. Ten days after surgery, a time when tandem fluorophore degradation was observed, ROS-producing cells were analyzed by flow cytometry (Fig. 3A). Although degradation of tandem fluorophores indicates the presence of increased ROS production it does not define which cell population is producing the elevated ROS. Thus, CellROX Deep Red dye was used in combination with nontandem conjugated Abs to further phenotype the ROS-producing cells (Fig. 3B). Notably, the frequency and number of ROS-producing cells was increased in splenocytes from CLP mice (Fig. 3C, 3F); the primary population of ROS-producing cells were Ly-6C^+^, suggestive of either monocytes or neutrophils (Fig. 3D, 3G). Conversely, T cells, which can produce free radicals during high metabolic activity, did not contribute to the increased ROS production (Fig. 3E, 3H); thus, myeloid cells are the predominate producers of ROS in the postseptic environment.

**FIGURE 4.**
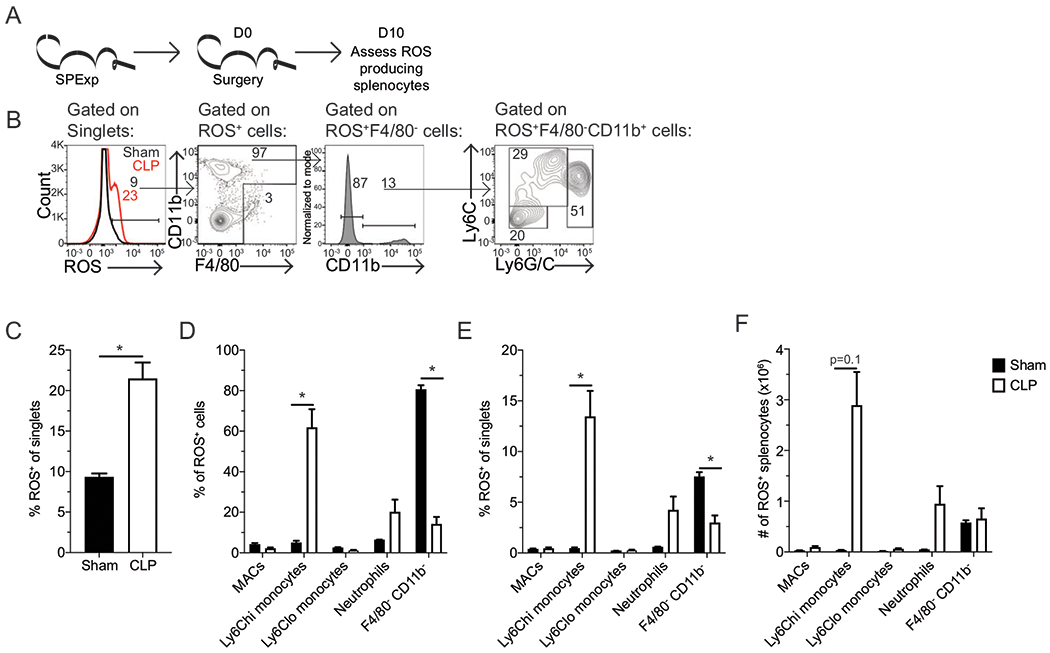
Persistent ROS production is by Ly-6C^hi^ monocytes. (**A**) Experimental design. SP_Exp_ mice were generated by infection with LCMV-Arm (2 × 10^5^ PFU i.p.) and then underwent sham or CLP surgery 30 d postinfection. ROS-producing splenocytes were assessed 10 d later by flow cytometry. (**B**) Representative gating of macrophages (MACs; F4/80^+^ cells), Ly-6C^hi^ monocytes (F4/80^−^, CD11b^+^, Ly-6G/C^lo^, Ly-6C^hi^), Ly-6C^lo^ monocytes (F4/80^−^, CD11b^+^, Ly-6G/C^lo^, Ly-6C^lo^), and neutrophils (F4/80^−^ CD11b^+^, Ly-6G/C^hi^, Ly-6C^hi^) among ROS-producing cells. Frequency (**C**–**E**) and number (**F**) of (C) ROS-producing cells, (D) MACs, Ly-6C^hi^ and Ly-6C^lo^ monocyte neutrophils, and undefined cells (F4/80^−^ CD11b^−^) among ROS-producing cells, and (E and F) ROS-producing MACs, Ly-6C^hi^ and Ly-6C^lo^ monocytes neutrophils, and undefined cells (F4/80^−^ CD11b^−^) among splenocytes. Data are representative of one experiment with three to four replicates per group. Error bars indicate SEM. *p < 0.05.

To further delineate the identity of the ROS-producing cells, additional flow cytometric profiling of SP_Exp_ mice was performed 10 d after sham or CLP surgery (Fig. 4A). Macrophages, Ly-6C^hi^ monocytes, Ly-6C^lo^ monocytes, and neutrophils were identified among the ROS-producing cells (Fig. 4B). The preponderance of the increase in ROS production (Fig. 4C) was among Ly-6C^hi^ monocytes, with a trending increase among neutrophils as well (Fig. 4D, 4E). These data suggest the majority of the Ly-6C^+^ cells contributing to the increased ROS production among CLP hosts are Ly-6C^hi^ monocytes.

### ROS-producing monocytes from CLP hosts are long lasting and have an immature phenotype

**FIGURE 5.**
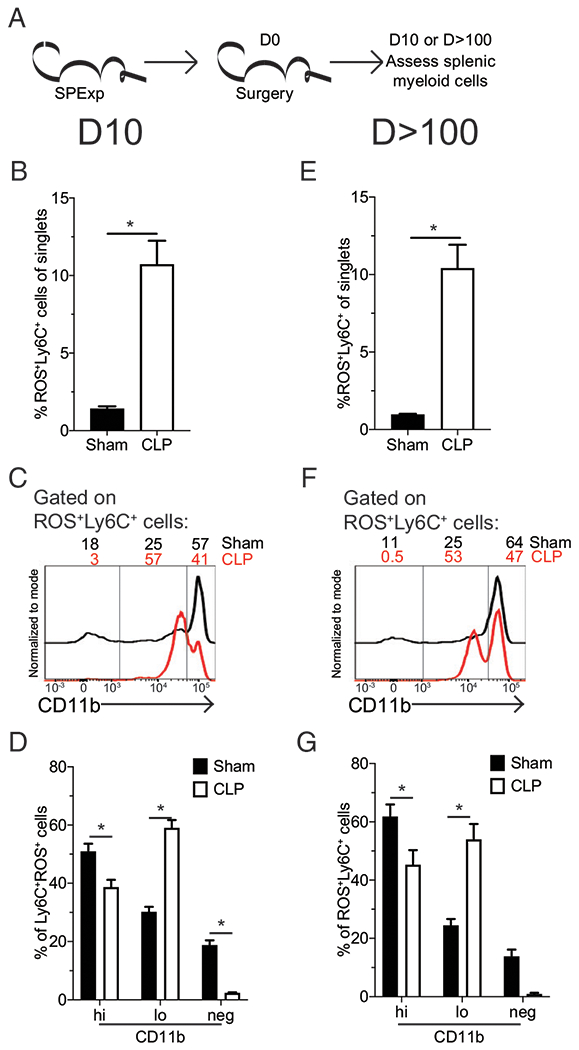
Sepsis leads to long-lasting ROS production by immature monocytes. (**A**) Experimental design. SP_Exp_ mice were generated by infection with LCMV-Arm (2 × 10^5^ PFU i.p.) and then underwent sham or CLP surgery 30 d later. ROS-producing splenocytes were assessed 10 or >100 d later by flow cytometry. 10 d postsurgery: (**B**) cumulative data of Ly-6C^+^ ROS-producing cells among total leukocytes; representative gating (**C**) and cumulative data (**D**) of CD11b expression by Ly-6C^+^ ROS-producing cells. More than 100 d postsurgery: (**E**) cumulative data of Ly-6C^+^ ROS-producing cells among total leukocytes. Representative gating (**F**) and cumulative data (**G**) of CD11b expression by Ly-6C^+^ ROS-producing cells. Data are representative of two repeated experiments with five replicates per group. Error bars indicate SEM. *p < 0.05.

Given that the sepsis-induced chronic immunoparalysis state is defined by both its extended duration and cellular recovery through emergency hematopoiesis, we interrogated the phenotype of these ROS^+^ Ly-6C^+^ cells both 10 and >100 d postsurgery (Fig. 5A). The increase in ROS-producing monocytes (Fig. 5B) observed in CLP hosts was attributed to an expansion in CD11b^lo^ monocytes (Fig. 5C, 5D). Splenic CD11b^lo^ monocytes are a less matured population of cells (57), and expansion of the CD11b^lo^ monocytes suggests this population arises as a result of emergency myelopoiesis following the septic-induced lymphopenia and impact on bone marrow progenitors (29). Valdes-Ferrer et al. (30) described a long-lasting population of phenotypically similar monocytes after sepsis. To address whether these cells were also contributing to ROS production late after sepsis, similar analysis was performed >100 d after surgery. Strikingly, the frequency of ROS-producing monocytes remained elevated at this extended timepoint post-CLP (Fig. 5E) because of a sustained increase in CD11b^lo^ ROS-producing monocytes (Fig. 5F, 5G). These data suggest the sepsis-induced change is highly durable, corresponding to a state of chronic immunoparalysis.

### Therapeutic vitamin C administration reduces sepsis disease severity and prevents sepsis-associated lasting ROS production

The persistent production of ROS observed potentially signifies a state of ongoing cellular stress. One of the stressors that can arise following a septic event is a vitamin C (ascorbate) deficiency similar to scurvy (58–60). Vitamin C serves as a donor antioxidant to reduce oxidizing free radicals and serves many other biochemical functions as a one-electron reducing agent (61, 62). Recently, clinical trials also indicate that therapeutic vitamin C administration may reduce early mortality (34, 40). Given the potential for vitamin C to limit the production of ROS, either through direct free radical reduction or indirect host support/limitation of cellular stress, its impact on ROS-producing cells was evaluated.

**FIGURE 6.**
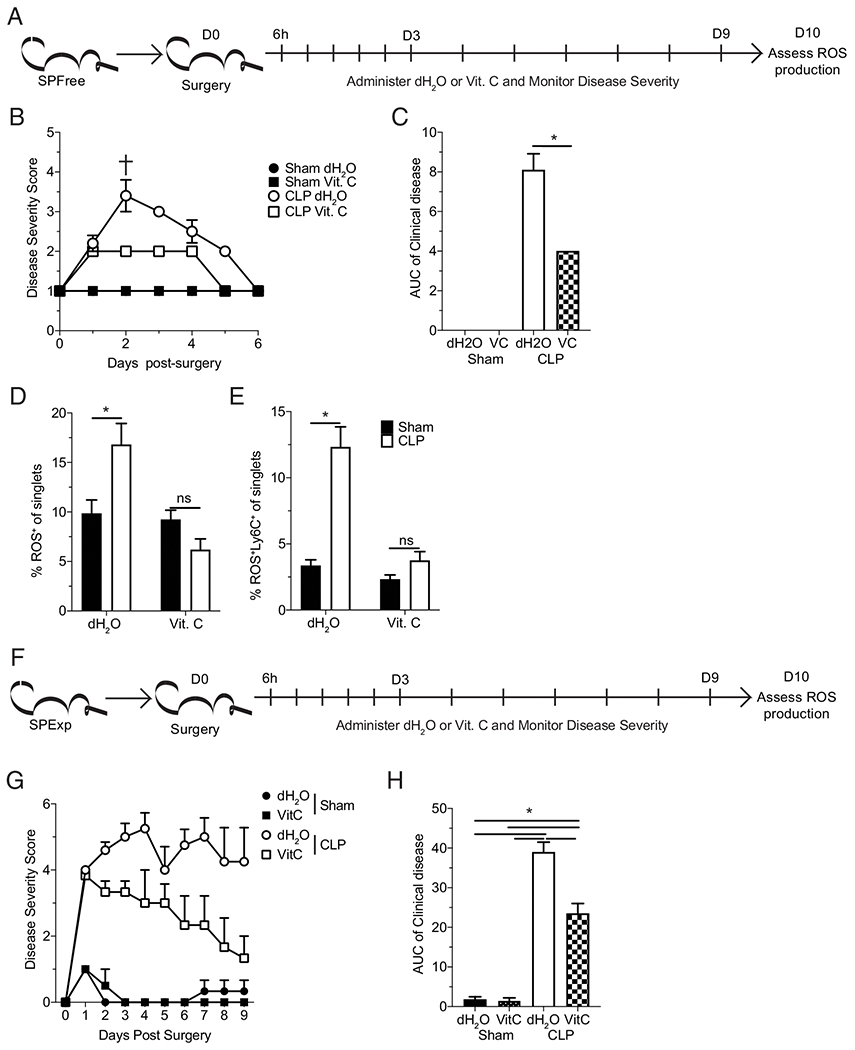
Therapeutic vitamin C administration reduces sepsis severity and prevents sepsis-associated ROS production. (**A**) Experimental design. SP_Free_ C57BL/6 mice underwent either sham or CLP surgery and then received 2.5 mg of vitamin C (Vit. C.; delivered i.p. in 100 μL dH_2_O i.p.)/25 g mouse beginning 6 h after surgery. Control mice received dH_2_O. Injections were repeated every 12 h for the first 3 d and then every 24 h for the subsequent 6 d. Disease severity was scored from 1–5 until recovery. ROS-producing splenocytes were assessed by flow cytometry 1 d after the last injection (10 d postsurgery). Average sepsis disease severity (**B**) for all experimental groups. The cross indicates the single mortality in dH_2_O-treated CLP mice. (**C**) Area under the curve of the disease scores for CLP experimental groups. Cumulative data of (**D**) ROS-producing cells and (**E**) Ly-6C^+^ ROS-producing cells among total leukocytes. (**F**) Experimental design. SP_Exp_ C57BL/6 mice underwent either sham or CLP surgery and then received 2.5 mg of vitamin C (Vit. C.; delivered i.p. in 100 μL dH_2_O i.p.)/25 g mouse beginning 6 h after surgery. Control mice received dH_2_O. Injections were repeated every 12 h for the first 3 d and then every 24 h for the subsequent 6 d. Disease severity was scored from 1–5 until recovery. ROS-producing splenocytes were assessed by flow cytometry 1 d after the last injection (10 d postsurgery). Average sepsis disease severity (**G**) for all experimental groups. Area under the curve (**H**) of the disease scores for CLP experimental groups. (B)–(E) are representative from two repeated experiments with 3-5 replicates per group and (F)–(H) are representative from a single experiment with 4–6 replicates per group. Error bars indicate SEM. The cross indicates mortality at the indicated timepoint, and the mouse was excluded from further scoring. *p < 0.05.

To assess the impact of vitamin C on ROS-producing monocytes as a potential clinically relevant therapeutic, we used dosages that were equivalent to those given to sepsis patients in recent clinical trials (34). A therapeutic administration strategy using i.p. administration was chosen, as vitamin C is not orally bioavailable at the concentrations supported by the i.v. trial injections but is achievable through i.p. injection (63, 64). Thus, the following dosing strategy was implemented: beginning 6 h after either sham or CLP surgery, SP_Free_ mice received either dH_2_O or vitamin C every 12 h for the first 3 d and then every 24 h for the subsequent 6 d. Readily available SP_Free_ mice were used to address the feasibility of therapeutic vitamin C treatment. ROS-producing cells were then assessed a day after the final dose (10 d postsurgery; Fig. 6A). CLP mice receiving vitamin C had significantly reduced disease severity, as assessed by clinical scoring, when compared with dH_2_O-treated animals (Fig. 6B, 6C), suggesting benefits to disease outcome can be seen/achieved early. Importantly, vitamin C treatment also reduced the frequency of ROS-producing cells and monocytes in particular (Fig. 6D, 6E). These data suggest vitamin C treatment may lead to a lasting benefit by reducing the frequency of ROS-producing monocytes. Based on the promising results observed in the SP_Free_ mice, we next sought to address whether this therapy also benefited the immunologically experienced hosts. Critically, when the same treatment strategy was used in SP_Exp_ mice (Fig. 6F), there was also a significant reduction in clinical disease severity (Fig. 6G, 6H).

In summary, these data demonstrate a beneficial effect of vitamin C treatment in reducing sepsis severity in immunologically naive and experienced hosts. The efficacy of this therapeutic strategy in hosts with differential immunologic experience may prove critical in the translational capability of this strategy. Further, this change in disease severity was observed early after sepsis induction and may translate into a reduction in the immunoparalysis state, thereby improving both long-term host immunity and survival.

### Elevated ROS production persists in patients who survive sepsis

**FIGURE 7.**
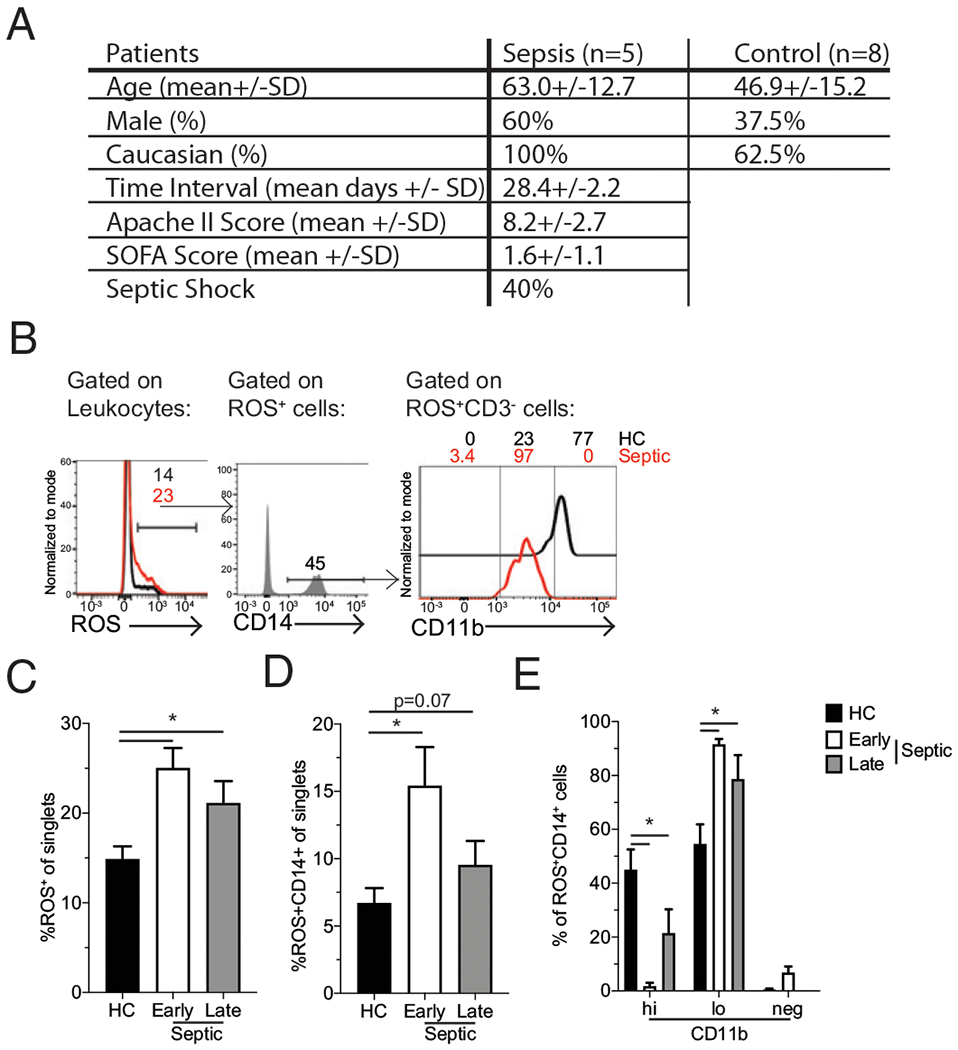
Sustained elevation in ROS production by immature monocytes from septic patients. (**A**) Patient demographics, Age, sex, ethnicity, sample time interval, acute physiology and chronic health evaluation II (APACHE II) score, sequential organ failure assessment (SOFA) score, and percentage of patients experiencing septic shock. (**B**) Representative gating of ROS-producing cells. Cumulative data of (**C**) ROS-producing cells and (**D**) CD14^+^ ROS-producing cells among total leukocytes. (**E**) Cumulative data of CD11b expression by CD14^+^ ROS-producing cells at early (<12 h after admission) and late (~28 d after initial collection) timepoints. Data are representative of one independent experiment with five to eight unique patients per group. Error bars indicate SEM. *p < 0.05.

Given that several disparities exist between mouse and human disease states, there is a strong need to translate findings from mice to humans to identify those findings with the most clinical relevance (56). Given the apparent relevance of these immature ROS-producing monocytes in our murine model, we sought to address both whether this population is also present and persists in septic patients. Thus, ROS-producing monocytes were examined in the peripheral blood of both septic patients and healthy controls. The “early” septic patient sample was taken within the first 12 h of admission, whereas the “late” samples were taken 30 plus or minus 5 d after the initial sample from the septic patients and compared with samples taken from healthy controls. We did not observe significant differences in demographic data between the septic patients and healthy controls (Fig. 7A). As anticipated, septic patients had severe disease, with 40% of septic patients developing septic shock. Notably, significant elevation in ROS production was present in early septic patient samples and persisted at the late timepoint (Fig. 7B, 7C). Similar to the mouse studies, the primary ROS producers were monocytes (CD14^+^ cells) (Fig. 7D) and was further associated with an increase in representation of CD11b^lo^ monocytes among ROS-producing cells (Fig. 7E). These data suggest that persistent ROS production by immature monocytes is a conserved feature of sepsis and may be relevant to the sepsis-induced immunoparalysis state.

## DISCUSSION

The sepsis-induced cytokine storm remains a critical hurdle in improving patient survival. However, if full recovery of patients is to be achieved, attention should also be placed on the lasting impact of sepsis. Our description of chronic production of ROS by immature splenic monocytes after CLP-induced sepsis extends previous reports describing sepsis-induced phenotypic changes in monocytes (30). This ROS production has the potential to impair lymphocyte function and thereby contribute to the immunoparalysis state seen in septic patients (56). Importantly, an increase in ROS production lasting ~28 d in samples from our septic patient cohort was observed, suggesting this has applicability for understanding clinical outcomes. These data parallel the observation that LPS-tolerized monocytes retain their capacity to produce ROS (65). Although our cohort of sepsis patients was small, the data obtained suggests the increase in ROS production may be a durable and conserved phenomenon. Future evaluation should use larger patient cohorts to determine how sepsis severity influences the magnitude and duration of ROS production by these immature monocytes. Additionally, there was a population on non-monocyte-producing ROS in septic patients, which may be neutrophils and another population that should be analyzed in more detail. Further, attention should also be given to whether the presence of ROS-producing CD11b^lo^ monocytes corresponds to enhanced suppression of lymphocyte functions as an index of sepsis-induced immunoparalysis. With respect to the cause of this ROS production, we have anecdotally observed that ROS production decreases with time after tissue harvest, suggesting that ROS production is maintained by environmental cues within the septic host. Future analyses delineating how well these monocytes subsequently respond to various triggering agents may help define how sepsis may be altering their overall function. If these cells are robust indicators of ongoing immunoparalysis, more targeted interventions to improve patient outcomes should be investigated.

A notable aspect regarding the reduced ROS production following vitamin C treatment is that early intervention conferred long-term benefit and possibly alleviates the chronic immunoparalysis state. The potential of vitamin C having both an early and lasting impact on decreasing sepsis severity and increased survival would be extremely important for a number of reasons. High-dose vitamin C treatment has demonstrated clinical efficacy in promoting tumor regression (62, 63, 66), suggesting the feasibility of a similar approach for septic patients. Despite the reasons for further investigation of vitamin C therapy for sepsis, some important counterpoints must also be discussed. In particular, the inability to achieve sufficient plasma concentration through oral administration means i.v. administration of vitamin C is the most feasible option to achieve the needed concentrations in patients (64). Because of the rapid clearance of high blood levels of vitamin C by the kidneys, patients may need to remain in the hospital to continue deriving its benefits (67). Moreover, long-term vitamin C administration would also require numerous i.v. injections or continuous i.v. delivery to maintain the elevated plasma levels needed to see a therapeutic effect. Special handling is also required because of the high rate at which vitamin C is oxidized. However, if the impact of vitamin C on the host is durable, then these treatment feasibility concerns would be substantially alleviated. Another complicating facet is that murine cells, unlike human cells, have the capacity to synthesize their own vitamin C, albeit achieving substantially lower concentrations in blood than what is achieved through therapeutic administration (63). This difference could explain some of the discordance seen between murine models and septic patients, which could also influence whether the response to vitamin C is durable in humans. When all of these points are taken in sum, the extent to which vitamin C treatment durably reduces ROS or alleviates aspects of sepsis-induced immunoparalysis should be addressed in future studies.

Alternately, it is unclear whether the benefit of vitamin C is derived solely through indirect mechanisms, such as reducing cellular stress, directly reducing ROS, or some combination thereof. Interrogation into these mechanisms may further promote additional therapeutic strategies. For instance, if the vitamin C–derived benefit is to some degree associated with reduction of free radicals, then more specific inhibitors of particular mechanisms of free radical generation can provide for targeted therapies to address this problem (68). Similarly, more robust characterization of the ROS-producing immature monocytes should be performed. This is made difficult by the proclivity of these cells to promote tandem fluorophore degradation (51). However, undertaking methods of reducing free radicals, such as addition of reducing agent to the staining buffer, allows for better clarity (51). Finally, understanding why this seemingly less mature population of cells persists may give insight into how sepsis influences progenitor populations to hamper subsequent immune responses.

The description of a long-lasting production of ROS by immature monocytes potentially demonstrates the durability of the immunoparalysis state. The persistence of this population likely reflects both how sepsis shapes the immune system of those individuals that survive the septic insult and how this alteration may feed back into the immunoparalysis state (e.g., ROS-mediated lymphocyte suppression) (26–28, 38). In addition, the presence of these cells in both mouse models and septic patients lends credence to the diligent use of animal models in the evaluation of septic insults (56). Thus, these data demonstrate the capacity to evaluate the chronic immunoparalysis state and how early interventions, such as vitamin C administration, may influence its development (34, 35, 58).

## Abbreviations used in this article:

AT: adoptive transfer
CLP: cecal ligation and puncture
LCMV: lymphocytic choriomeningitis virus
LCMV-Arm: LCMV-Armstrong
ROS: reactive oxygen species
SP_Exp_: specific pathogen–experienced
SP_Free_: specific pathogen–free
